# Comparative Evaluation of Antioxidant and Antidiabetic Activities of ZrO_2_ and MgO Nanoparticles Biosynthesized from Unripe *Solanum trilobatum* Fruits: Insights from In Vitro and In Silico Studies

**DOI:** 10.3390/nano15171372

**Published:** 2025-09-05

**Authors:** Kumaresan Rathika, Periyanayagam Arockia Doss, John Rose Arul Hency Sheela, Velayutham Gurunathan, K. J. Senthil Kumar, Chidambaram Sathishkumar, Vediyappan Thirumal, Jinho Kim

**Affiliations:** 1 Department of Chemistry, Muslim Arts College (Affiliated to Manonmaniam Sundaranar University, Thirunelveli), KanyaKumari 629174, Tamil Nadu, India; rathika.jothi81@gmail.com (K.R.); arulhencysheela@gmail.com (J.R.A.H.S.); 2 Department of Chemistry, St. Joseph’s College (Autonomous), Tiruchirappalli 620002, Tamil Nadu, India; nivedoss@gmail.com; 3 Research Department of Chemistry, Bishop Heber College (Affiliated to Bharathidasan University), Tiruchirappalli 620017, Tamil Nadu, India; v.gurunathan.v@gmail.com; 4 Center for General Education, National Chung Hsing University, Taichung 402, Taiwan; zenkumar@dragon.nchu.edu.tw; 5 Department of Biotechnology, Saveetha Institute of Medical and Technical Sciences, Saveetha University, Thandalam, Chennai 602105, Tamil Nadu, India; 6 Nextgen Academic Research, Perambalur 621107, Tamil Nadu, India; 7 Department of Mechanical Engineering, Yeungnam University, Gyeongsan-si 38541, Gyeongbuk-do, Republic of Korea

**Keywords:** antidiabetic, antioxidant, alpha-amylase, docking, magnesium oxide, *Solanum trilobatum*, Zirconium dioxide

## Abstract

Herbs offer people not just sustenance and housing but also serve as a key supplier of pharmaceuticals. This research is designed to assess the antioxidant and antidiabetic properties of green-produced zirconium dioxide and magnesium oxide nanoparticles (ZrO_2_ and MgO NPs) utilizing extracts from unripe *Solanum trilobatum* fruit. ZrO_2_ and MgO NPs have garnered considerable interest owing to their superior bioavailability, lower toxicity, and many uses across the healthcare and commercial industries. Scientific approaches, such as diverse spectroscopic and microscopic approaches, validated the creation of agglomerated spherical ZrO_2_ and MgO NPs, measuring between 15 and 30 and 60 and 80 nm, with a mixed-phase composition consisting of monoclinic and tetragonal phases for ZrO_2_ and a face-centered cubic structure for MgO NPs. UV–vis studies revealed a distinct peak at 378 and 290 nm for ZrO_2_ and MgO NPs, suggesting efficient settling through the phytonutrients in *S. trilobatum*. The antioxidant capacity of ZrO_2_ and MgO NPs was evaluated utilizing DPPH and FRAP reducing power assays. The diabetic effectiveness of ZrO_2_ and MgO NPs was examined by alpha-amylase and alpha-glucosidase assays. The optimum doses of 500 and 1000 μg/mL were shown to be efficient in reducing radical species. Green-produced ZrO_2_ and MgO NPs exhibited a dose-dependent reaction, with greater amounts of ZrO_2_ and MgO NPs exerting a more pronounced inhibitory effect on the catalytic sites of enzymes. This work suggests that ZrO_2_ and MgO NPs may attach to charge-carrying entities and function as rival inhibitors, therefore decelerating the enzyme–substrate reaction and inhibiting enzymatic degradation. Molecular docking analysis of ZrO_2_ and MgO NPs with three proteins (2F6D, 2QV4, and 3MNG) implicated in antidiabetic and antioxidant studies demonstrated the interaction of ZrO_2_ and MgO NPs with the target proteins. The results indicated the in vitro effectiveness of phytosynthesized ZrO_2_ and MgO NPs as antidiabetic antioxidant agents, which may be used in the formulation of alternative treatment strategies against diabetes and oxidative stress. In summary, the green production of ZrO_2_ and MgO NPs with *Solanum trilobatum* unripe fruit extract is an efficient, environmentally sustainable process that yields nanomaterials with significant antioxidant and antidiabetic characteristics, underscoring their prospective uses in biomedical research.

## 1. Introduction

Diabetes mellitus is a leading ‘illness of society’ that is widespread worldwide and influences a significant portion of the people worldwide. The prevalence of diabetes is elevated, posing an ongoing problem for worldwide medical systems. The anticipated number of cases is predicted to be 592 million by 2040 [1]. The condition, including those of Type 1 and Type 2, is primarily characterized by diminished insulin production or insulin resistance, resulting in significant disruptions in glucose levels and lipid and protein metabolism, along with an increase in blood sugar levels [2]. Hyperglycemia is related to chronic medical problems such as heart disease, kidney problems, hepatic diseases, and peripheral neuropathy, which refers to damage to the nerves across the body, especially in the extremities. As a result, it induces diabetic blisters (including heel and ankle wounds) that adversely impact the wound’s healing by diminishing the supply of oxygen and blood to the affected region. Furthermore, it leads to a diminished immune response, which reduces the nutrient supply necessary for wound recuperation and restoration [3]. Glycemic management is regarded as the fundamental aspect of diabetic mellitus treatment. Realistically accessible antidiabetic medications are frequently employed to mitigate hyperglycemia. Nevertheless, several hazards linked to diabetes and the negative consequences of these medications have redirected research focus towards the exploration of innovative plant-derived antidiabetic agents [4,5]. Therefore, it is crucial to identify alternative medications outside of pharmaceutical options.

The development of nanotechnology encompasses the development, fabrication, characterization, and management of substances within the nanoscale that spans 1 to 100 nanometers. It involves the alteration of materials at molecular and atomic scales. The use of nanostructures is increasing daily. Biomedical studies use the distinctive characteristics of nanostructures [6,7]. Nanomaterials are beneficial for both in vivo and in vitro healthcare studies as well as uses, including diagnostics and healing goals [8,9]. Understanding of metabolic processes and the impact of nanostructures on humans is limited. Therefore, it is essential to understand the relationship between nanomaterials and living things. The nanoparticle production approach is neither safe nor environmentally conscious. Consequently, the environmentally friendly approach to nanoparticle production is used [10,11]. Recently, scientists have concentrated on using botanicals for the production of nanomaterials because of their environmentally favorable characteristics and economic efficiency. Biogenesis is devoid of elevated temperatures, significant energy, or hazardous substances. Plants generate more stable nanoparticles, and the likelihood of contaminants is reduced relative to various artificial techniques [12]. The brief manufacturing duration, safety, low cost, and enhanced quantity of manufacturing augment the utilization of phytonutrients in NP creation. Moreover, herbal resources include many phytonutrients and metabolites that enhance their biotic action [13].

*S. trilobatum*, referred to as Thuthuvalai in Tamil, is a therapeutic herb within the family Solanaceae. Investigations in this domain have garnered significant interest due to the increasing focus on the role of free radicals and oxidative damage in the development of illnesses [14,15,16]. Leafy greens offer an important supply of vitamins and minerals, including crude fiber, iron, fats, phosphorus, carbs, and calcium. This plant is utilized for the treatment of coughs, common colds, and asthma [17,18,19]. This plant was employed in the Ayurvedic medicinal tradition to address breathing problems, chronic feverish diseases, tuberculosis, and afflictions of the cardiovascular system and liver. This plant has anticancer, antibacterial, antimitotic, and antimicrobial activities [20,21].

ZrO_2_ NPs possess distinct characteristics, including elevated chemical and thermal stability, facilitating their application in numerous sectors. They are utilized in refractory materials, fuel cells, sensors, thermal barrier coatings, and as cutting instruments. ZrO_2_ is a noteworthy metal oxide for investigation, particularly in medical and health fields, due to its great thermal stability and resistance to microbiological and chemical agents. Its uses include ligament prostheses, fillers in paints, and dental bridges and crowns [13].

The use of trace elements present in the human body, such as copper, zinc, magnesium, selenium, and iron, in the creation of nanoparticles is now being explored in the domain of nanomedicine [22]. These nanomaterials are regarded as alternative possibilities owing to their distinctive plasmonic and optoelectronic capabilities. Magnesium nanoparticles have garnered attention for several uses, including catalysis, adsorption, drug administration, antibacterial use, bone implantation, and diabetes therapy [23,24,25]. Magnesium, a non-metal, is a vital nutrient necessary for the optimum functioning of the brain and body. Essential biological processes such as hemostasis, cardiac rhythm, and muscle contraction are regulated by magnesium [26]. Prior research has emphasized the connection between magnesium and diabetes, since individuals with type 2 diabetes often have decreased blood magnesium levels. Magnesium modulates insulin activity, particularly the post-receptor mechanisms associated with insulin-mediated glucose uptake and other enzymatic activities in glycolysis [27,28]. Magnesium’s role in glucose and insulin metabolism has been established, and magnesium supplementation has been shown to reduce glycemic response in individuals with type 2 diabetes [29]. The use of a magnesium source may be advantageous in preventing and treating type 2 diabetes.

This work involves the synthesis of therapeutically beneficial ZrO_2_ and MgO NPs utilizing a medicinal plant of considerable importance in healthcare. The crystalline, biochemical, and structural properties of phytofabricated ZrO_2_ and MgO NPs were analyzed using UV–vis, SEM, XRD, TEM, and FT-IR techniques. Ultimately, antioxidant and antidiabetic evaluations were conducted to delineate the therapeutic attributes of the ZrO_2_ and MgO NPs.

## 2. Materials and Methods

Unripe fruit of *Solanum trilobatum* was harvested within the rural regions of Namakkal, Tamil Nadu, India. ZrOCl_2_⋅8H_2_O and MgSO_4_ were acquired from Sigma-Aldrich Chemicals, Bangalore, India. All compounds obtained are of analytical grade and utilized without additional purification. Across the course of the study, double-distilled water was employed.

### 2.1. Preparation of Leaf Extract

Fresh, unripe berries of *Solanum trilobatum* were repeatedly cleaned in distilled water to eliminate exterior dirt. Initially, 10 g of unripe fruits were chopped into tiny fragments and mixed with 100 mL of deionized water while being continuously heated at 90 °C for 30 min, as long as it took for the color to be transformed to a light brown hue. The resulting solution was ultimately filtered using Whatman filter paper No. 1.

### 2.2. Biogenesis of ZrO_2_-NPs

To synthesize ZrO_2_ NPs, 10 mL of *S. trilobatum* extract was incrementally infused into 10 mL of a 0.1 M aqueous ZrOCl_2_⋅8H_2_O solution while maintaining constant mixing at 75 °C for 3 to 4 h. One milliliter of 10% NaOH solution was added to adjust the pH of the reaction mixture. The samples were cleaned by centrifuging at 6000 rpm for 10 min and then washed with double-distilled water to eliminate impurities. The synthetic material was dehydrated in an oven at 100 °C. The final residue was subsequently calcined at 600 °C to yield powder (ZrO_2_-NPs), which was kept for additional investigation.

### 2.3. Synthesis of MgO NPs

To synthesize MgO NPs, 10 mL of *S. trilobatum* extract was incrementally infused into 10 mL of a 0.1 M aqueous MgSO4 solution while maintaining constant mixing at 75 °C for 3 to 4 h. One milliliter of 10% NaOH solution was added to adjust the pH of the reaction mixture. The samples were cleaned by centrifuging at 5000 rpm for 5–7 min and then washed with deionized water to eliminate impurities. The synthetic material was dried out in an oven at 70 °C. The dried specimen was subsequently calcined in a muffle furnace at 800 °C to yield powder (MgO NPs), which was further investigated.

### 2.4. Characterization of Phytofabricated ZrO_2_-NPs

The structure and form of phytofabricated ZrO_2_ and MgO were analyzed employing a SHIMADZU 6000 X-Ray diffractometer with CuKα radiation (k = 1.5406 Å). The visible spectrum was obtained using a JASCO V-670 spectrometer within the range of 300–800 nm. The surface morphology, elemental formulations, and crystalline size determination of the material were analyzed with scanning electron microscopy (SEM) utilizing a Hitachi S-4500, supplemented by energy dispersive X-ray (EDX) spectrometry. FT-IR spectra were acquired with an FT-IR (FT/IR-4600 type A D044761786).

### 2.5. Antioxidant Activity

#### 2.5.1. DPPH Radical Scavenging Assay

The ability of antioxidants to easily donate hydrogen atoms is believed to be the reason underlying their efficiency in scavenging DPPH radicals. Radical scavenging actions signify a crucial role in reducing the harmful impact of free radicals in several illnesses, like diabetes. An assessment was conducted on plant extracts and ZrO_2_ and MgO NPs to determine their efficacy in scavenging DPPH radicals using an altered version of a recognized technique [29]. Two milliliters of different concentrations (ranging from 125 to 1000 μM) of the plant extract, ZrO2 and MgO NPs, or the control (ascorbic acid) were mixed into 2 mL of a 0.3 mM DPPH solution in CH_3_OH. The combination was agitated and sealed in a dimly lit container at ambient temperature (25 °C) for 30 min. Thereafter, the absorbance was analyzed at a wavelength of 516 nm, and the efficacy of DPPH radical scavenging was evaluated employing the resulting calculation:

DPPH Scavenging activity (%) = (1 − Absorbance of compound/Absorbance of control) × 100

#### 2.5.2. FRAP Assay

The FRAP of the plant extract and ZrO_2_ and MgO NPs was evaluated using the usual approach outlined in other studies, alongside slight adjustments [29]. An aqueous solution of the components, with concentrations ranging from 125 to 1000 μM, was combined with 0.75 mL of K_4_Fe(CN)_6_ (1% *w*/*v*) and 0.75 mL of phosphate buffer (0.2 M, pH 6.6). The mixture then underwent incubation at 50 °C for 20 min. The reaction was terminated by adding 0.75 mL of a 10% CCl_3_COOH acid solution. The entire combination was centrifuged at 8000 rpm for 10 min. The fluid portion was then combined with 0.1 mL of FeCl_3_ (0.1%, *w*/*v*) and 1.5 mL of water. An optical spectrometer determined the solution’s absorbance at 700 nm.

### 2.6. Antidiabetic Studies

#### 2.6.1. α-Glucosidase Inhibition Activity

The enzymatic inhibition properties of the plant extract and ZrO_2_ NPs on α-glucosidase were assessed. α-glucosidase catalyzes the conversion of disaccharides to monosaccharides, leading to postprandial hyperglycemia. Consequently, inhibitors of α-glucosidase are efficacious in controlling hyperglycemia by postponing the breakdown of carbohydrates, thus reducing the likelihood of developing diabetes and other illnesses associated with carbohydrates. The antidiabetic effect of ZrO_2_ and MgO NPs was assessed by adapting a pre-existing approach [30]. Initially, 50 μL of plant extract and ZrO_2_ and MgO NPs at varying dosages (125–1000 μM) were introduced to a phosphate buffer (pH 6.8, 100 mM) containing a corresponding concentration of α-glucosidase (1.0 U/mL) at ambient temperature for 15 min. The resultant solution was then amalgamated with a 100 μL solution of 5 mM p-nitrophenyl-α-D-glucopyranoside (pNPG) in phosphate buffer (100 mM, pH 6.8). The mixture was subsequently incubated at 37 °C for 20 min. Acarbose, a standard medication, served as the positive control. The absorbance of the liberated p-nitrophenol was assessed at 405 nm, and the inhibitory effect was quantified as a percentage in relation to the experimental control without inhibiting agents, as described by the following equation:

% inhibition = [(Absorbance of control − Absorbance of sample)/Absorbance of control] × 100.

#### 2.6.2. α-Amylase Inhibition Activity 

The inhibitory effect of the target compounds on α-amylase was evaluated using the technique designated in other investigations, with slight modifications [31]. The entire amount of 50 μL of progressively increasing dosages (125–1000 μM) was combined with equal amounts of porcine pancreatic amylase (2 U/mL) in phosphate buffer (pH 6.8, 100 mM) for 10 min at room temperature. Additionally, 50 μL of a 0.5% starch solution in phosphate buffer (pH 6.8, 100 mM) was introduced to the resultant solution and incubated at 37 °C for 10 min. Afterwards, 100 μL of dinitro salicylate (DNS) coloring agent was included in the resultant solution and heated for 10 min. Acarbose served as the reference medication. Subsequently, the absorbance was recorded at 540 nm, and the inhibitory effect was determined as a percentage of the standard, employing a straightforward calculation:

% inhibition = [(Absorbance of control − Absorbance of sample)/Absorbance of control] × 100.

### 2.7. Molecular Docking Methods

#### 2.7.1. Ligand and Protein Preparation

The ZrO_2_, MgO, and the controls, acarbose and ascorbic acid, were modeled utilizing the Chem3D Pro 12.0 and ChemDraw Ultra 12.0 programs. The subsequent structures have been conserved in a PDB file for a docking experiment. The present investigation examined the Protein Data Bank (PDB) to obtain the crystallographic structures of receptors for docking purposes. The crystalline structure of glucoamylase combined with acarbose (PDB ID: 2F6D) was utilized for the α-glucosidase inhibition experiment. The three-dimensional structure of human pancreatic α-amylase combined with acarbose and nitrile (PDB ID: 2QV4) was employed for the α-amylase inhibition experiment. This work used the crystalline structure of a human antioxidant enzyme with the co-crystallized inhibitor DTT (PDB ID: 3MNG) to investigate its antioxidant capacity. In each crystalline form, a single protein chain was chosen and preserved as the dock receptor, whereas heteroatoms, other ligands, other chains, and water molecules were removed. The inbound ligand in each crystalline structure was defined at a distance of less than 5 Å. The configuration of amino acids in this area was recorded and considered the limits of the binding pocket for the docking experiment.

#### 2.7.2. Docking Assessment

The docking investigations were carried out using the Autodock Vina tool [32]. In each instance of a biological assessment, the protein structure was first entered into Vina and then transformed into a macromolecule by converting it into a pdbqt for docking applications. The search grid for the glucoamylase protein (PDB ID: 2F6D) is centered at x-coordinate 10.945, y-coordinate 8.351, and z-coordinate −9.511. The grid consists of the parameters: size_x, y, z = 24, 24, 24. The grid maintains a uniform spacing of 1.0 Å between each point. The focal coordinates of the search grid for the α-amylase protein (PDB ID: 2QV4) are x: 10.721, y: 48.233, and z: 20.805. The grid dimensions were defined as x, y, z = 24, 24, 24, with a spacing of 1.0 Å. The human antioxidant enzyme search grid (PDB ID: 3MNG) is located at coordinates x: 8.341, y: 45.637, and z: 30.621. The grid dimensions are x, y, z = 18, 18, 18, and have a spacing of 1.0 Å. A positive integer value of 8 has been assigned to the exhaustiveness level. The other variables were set to their usual settings for Vina docking and were not specifically stated. The chemical with the smallest binding affinity score is considered to possess the highest activity. The docking results were visually evaluated using the Discovery Studio 2019 software.

## 3. Results and Discussion

### 3.1. UV–Visible Analysis

**Figure 1:**
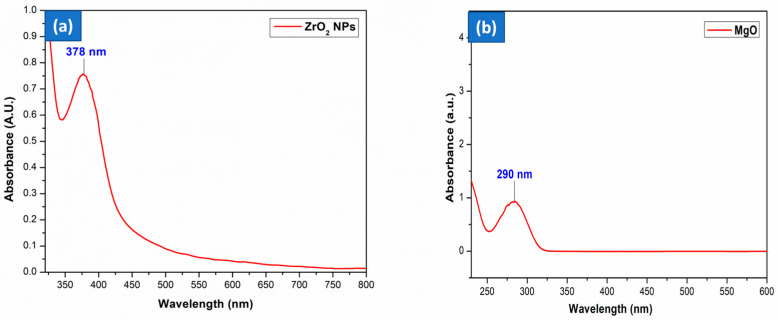
UV–visible spectra of (**a**) ZrO_2_; (**b**) MgO NPs.

Figure 1a displays the UV–visible spectrum of phytofabricated ZrO_2_ and MgO NPs. The dispersion concentrations of a stock solution (0.5 mg.mL^−1^) were prepared for the formation of the ZrO_2_ nanostructure, as evident from the (Zr^4+^ and O^2−^) reduction and stabilization [33]. The fabricated ZrO_2_ NPs showed significant absorbance at 378 nm. For ZrO NPs, the SPR spectra peak ranges from 250 to 400 nm due to the charge transition from oxide species to Zr cation (O^−^→Zr^4+^) [34]. A comparable outcome was seen for ZrO_2_ NPs employing *Ficus benghalensis* leaf extract [34]. The outcome indicates a greater contribution from the spectrum shown in the absorption area. ZrO_2_ NPs may possess a greater number of surface flaws owing to their elevated surface area [35].

A progressive color change from pale green to pale white in the reaction mixture provided visual evidence of Mg^2+^ ion reduction to MgO NPs by the unripe fruit extract of *S. trilobatum*. As seen in Figure 1b, the optical characteristics of the MgO nanoparticles were recorded using UV–visible spectroscopy across the 200–600 nm range, as depicted in Figure 1b. In the UV–visible spectroscopy, the sharp absorbance peak at 290 nm indicated the formation of small-sized particles of MgO NPs. The absorption peak for MgO prepared by leaf extracts of *Mangifera indica*, *Azadirachta indica*, and *Carica papaya* was observed at 291, 288, and 288 nm, respectively [36]. The green synthesized NPs usually demonstrate a surface plasmon resonance, which leads to absorption in the UV–vis region with distinctive optoelectronic properties.

### 3.2. FT-IR Studies

**Figure 2:**
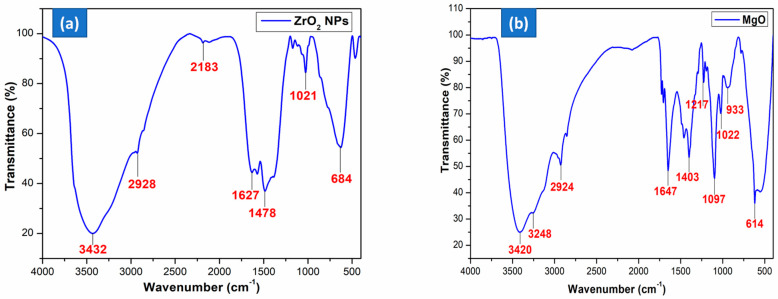
FT-IR spectrum of (**a**) ZrO_2_; (**b**) MgO NPs.

An FT-IR examination was conducted to classify the functional components contained in the produced ZrO_2_ NPs, as seen in Figure 2a. The significant peak at 3432 cm^−1^ correlates to the O–H moiety due to moisture adsorbed on the material’s surface [37]. The band at 1478 cm^−1^ corresponds to vibrations resulting from C=O stretching. The absorption peak in the range of 684 cm^−1^ to 1021 cm^−1^ is indicative of a Zr–O–Zr bond, hence validating the creation of crystalline ZrO_2_ NPs [37,38]. This suggests that the creation of ZrO_2_ NPs likely entails the involvement of amines, carboxyl moieties, intermediates of phenolic assemblies, carbohydrates, and proteins from unripe fruit extract during the reduction steps.

FT-IR spectroscopy provided insights into the functional groups associated with the synthesized MgO nanoparticles, highlighting phenolic signatures and metal–ligand interactions. Figure 2b shows the spectral data of MgO NPs synthesized using *S. trilobatum* unripe fruit extract. The broad absorption at 3420 cm^−1^ reflects O–H stretching vibrations, confirming the presence of hydroxyl groups [39]. The band at 2924 cm^−1^ signifies C–H stretching from aromatic aldehydes. The peak at 1647 cm^−1^ indicates the presence of carbonyl (C=O) functionalities, while the 1097 cm^−1^ band suggests C–O bond stretching, pointing to saturated primary alcohol groups [40]. A band at 1403 cm^−1^, associated with –CH_2_ bending, suggests the existence of aromatic tertiary amines or carbonyl-containing structures such as aldehydes and ketones. The characteristic Mg–O vibration at 614 cm^−1^ confirms the presence of MgO nanoparticles.

### 3.3. SEM, EDX, and TEM Analysis

**Figure 3:**
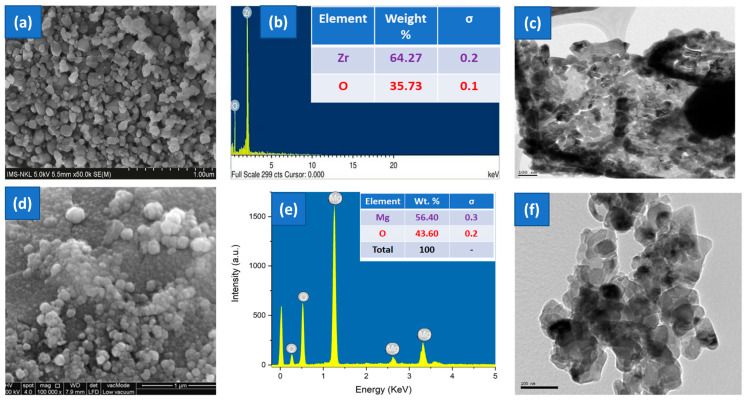
(**a**,**d**) SEM, (**b**,**e**) EDX, and (**c**,**f**) TEM images of ZrO_2_ and MgO NPs.

The surface morphology of ZrO_2_ NPs was examined by SEM, as seen in Figure 3a. The ZrO_2_ NPs have a spherical morphology with little aggregation of particles. The elemental makeup of ZrO_2_ NPs was assessed using EDX spectroscopy. Zirconium and oxygen atoms were indicated by bands at 0.5 and 2.0 keV, respectively. The weight percentages of zirconium and oxygen atoms were 64.27% and 35.73%, respectively. Figure 3b shows the EDX spectra of ZrO_2_ NPs. Figure 3c illustrates morphological investigations performed by TEM examination. The predominant particles detected were round and showed agglomeration. The mean size of the particles was approximated to be between 15 and 30 nm, exhibiting a spherical morphology.

SEM analysis was conducted to examine the morphological features and particle size of the synthesized MgO nanoparticles. As illustrated in Figure 3d, the particles exhibited a predominantly spherical morphology with slight agglomeration. The nanoparticles appeared as discrete entities, evenly distributed across the surface. EDX elemental analysis (Figure 3e) revealed a strong magnesium signal averaging 56.40% and an oxygen signal at 43.60%. Peaks observed at 1.3, 2.6, and 3.3 keV correspond to magnesium, whereas oxygen was detected at 0.3 and 0.5 keV. Figure 3f illustrates the formation of uniformly distributed nanoparticles with consistent diameters between 60 and 80 nm. The spherical and uniform grain structure observed in TEM micrographs corroborates the formation of highly crystalline MgO nanoparticles.

### 3.4. XRD Analysis

**Figure 4:**
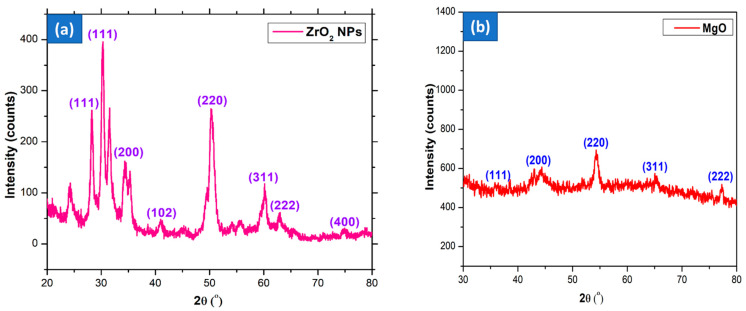
XRD pattern of biosynthesized (**a**) ZrO_2_; (**b**) MgO NPs.

The XRD structure of the phytofabricated ZrO_2_ NPs is displayed in Figure 4a. The principal diffraction peaks are seen at 2θ = 29.41°, 30.45°, 35.69°, 41.76°, 50.65°, 60.80°, 62.13°, and 75.39°, correspond to the (1 1 1), (1 1 1), (2 0 0), (1 0 2), (2 2 0), (3 1 1), (2 2 2), and (4 0 0) diffraction patterns, accordingly (JCPDS Card No: 37-1484 and 42-1164) [34]. This suggests that the generated ZrO_2_-NPs exist in both tetragonal and monoclinic crystallinity.

D = 0.9*λ*/*β*cos*θ* (1)

where β denotes the full width at half maximum of the peak, λ signifies the X-ray wavelength of CuKα radiation, and D represents the crystalline size. The mean crystallite size of ZrO_2_ NPs was 26.74 nm. A comparable crystalline size was achieved by producing ZrO_2_ NPs employing Moringa oleifera leaf extract [41].

The crystalline characteristics, particle dimensions, and phase integrity of the synthesized magnesium oxide nanoparticles were determined using powder X-ray diffraction (PXRD). The diffraction pattern obtained from MgO nanostructures synthesized using *S. trilobatum* unripe fruit extract revealed characteristic peaks corresponding to the (111), (200), (220), (311), and (222) crystal planes. These reflections match those of the JCPDS file No. 87–0653, indicating a polycrystalline face-centered cubic structure. The presence of additional diffraction signals is likely due to bioorganic compounds from the plant extract, which are known to influence the nanoparticle’s morphology, structural coherence, and phase composition. The crystallite size of MgO prepared by the *S. trilobatum* plant extract was calculated using the Debye Scherrer equation. The biosynthesis yields MgO NPs with a particle size of 78 nm. The PXRD spectra of MgO NPs were presented in Figure 4b.

### 3.5. Antioxidant Assessment

#### 3.5.1. DPPH Radical Scavenging Assay

Table 1 presents the DPPH scavenging characteristics of the plant extract and ZrO_2_ NPs. The plant extract and ZrO_2_ and MgO NPs exhibited enhanced DPPH scavenging capacity at concentrations between 125 and 500 μM. However, the control exhibits superior activity compared to the plant extract at a greater concentration of 1000 μM. Equally, the capacity of plant extract and ZrO_2_ and MgO NPs to scavenge DPPH was enhanced with higher concentrations. The DPPH scavenging capacity exhibited a dose-dependent pattern. Compared to the plant extract (IC_50_ = 323.49 μM), the ZrO_2_ and MgO NPs showed a higher DPPH scavenging capacity (IC_50_ = 191.96 and 185.23 μM). Likewise, the plant extract and MgO NPs proved DPPH quenching characteristics; however, the MgO NPs showed a higher scavenging rate (IC_50_ = 185.23 μM), signifying enhanced activity relative to the control.

#### 3.5.2. FRAP Antioxidant Assay

Table 2 indicates that the relative FRAP antioxidant ability of the plant extract and ZrO_2_ and MgO NPs, in association with the standard ascorbic acid, demonstrates that the plant extract exhibits inferior ferric reducing antioxidant effects relative to the ZrO_2_ and MgO NPs. Likewise, the ferric reducing antioxidant characteristics of the plant extract and ZrO_2_ and MgO NPs were enhanced with higher concentrations. The antioxidant activities of ferric reducing agents exhibited a dose-dependent pattern. Compared to the plant extract (IC_50_ = 560.08 μM), the ZrO_2_ and MgO NPs exhibited substantial ferric reducing antioxidant capabilities (IC_50_ = 161.86 and 143.12 μM). Furthermore, the MgO and ZrO_2_ NPs exhibited ferric reducing antioxidant characteristics. The MgO NPs have a greater scavenging rate (IC_50_ = 143.12 μM), indicating superior activity compared to the control ascorbic acid (IC_50_ = 223.51 μM).

### 3.6. Antidiabetic Activity

#### 3.6.1. α-Glucosidase Inhibition Action

The antidiabetic activities of plant extract and synthesized ZrO_2_ and MgO NPs were assessed through an α-glucosidase inhibition assay. The compound acarbose was used as a standard. At 1000 μM, the tested substances displayed a higher inhibition rate. The α-glucosidase inhibition assay followed a dose-dependent pattern. The plant extract displayed moderate α-glucosidase inhibition ability compared to the synthesized ZrO_2_ and MgO NPs. The synthesized ZrO_2_ and MgO NPs (IC_50_ = 155.76 and 138.84 μM) exhibited notable inhibitory action towards α-glucosidase in comparison to the plant extract (IC_50_ = 475.92 μM). Related to the control acarbose with an IC_50_ value of 256.27 μM, the MgO NPs with an IC_50_ value of 138.84 μM exhibited substantial antidiabetic action. Only the plant extract exhibited reduced antidiabetic potential, with an IC_50_ value of 475.92 μM, related to the control acarbose (IC_50_ = 256.37 μM). The findings are represented in Table 3.

#### 3.6.2. α-Amylase Inhibitory Action

In Table 4, the α-amylase inhibitory effects of the plant extract and ZrO_2_ and MgO NPs are displayed. The obtained result demonstrates that the ZrO_2_ and MgO NPs exhibit greater potency than the plant extract. However, the inhibitory action of plant extract and ZrO_2_ and MgO NPs is dependent on concentration. The ZrO_2_ and MgO NPs exhibited superior antidiabetic action (IC_50_ = 145.95 and 129.45 μM) compared to the plant extract (IC_50_ = 378.78 μM) and the control acarbose (IC_50_ = 225.65 μM). The plant extract had a weaker antidiabetic potential, as indicated by its IC_50_ value of 378.78 μM, than the control acarbose, which had an IC_50_ value of 225.65 μM. Nevertheless, the efficacy of the plant extract in treating diabetes was enhanced when combined with zirconium and magnesium metal ions.

## 4. Docking Studies

### 4.1. Antidiabetic

#### 4.1.1. α-Glucosidase Inhibition

**Figure 5:**
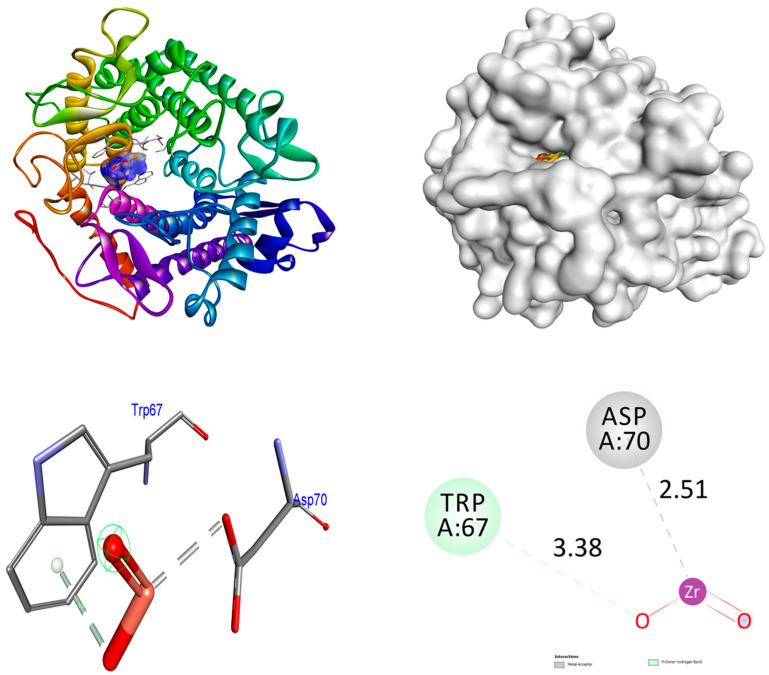
Interactions of ZrO_2_ NPs inside the binding cavity of 2F6D.

**Figure 6:**
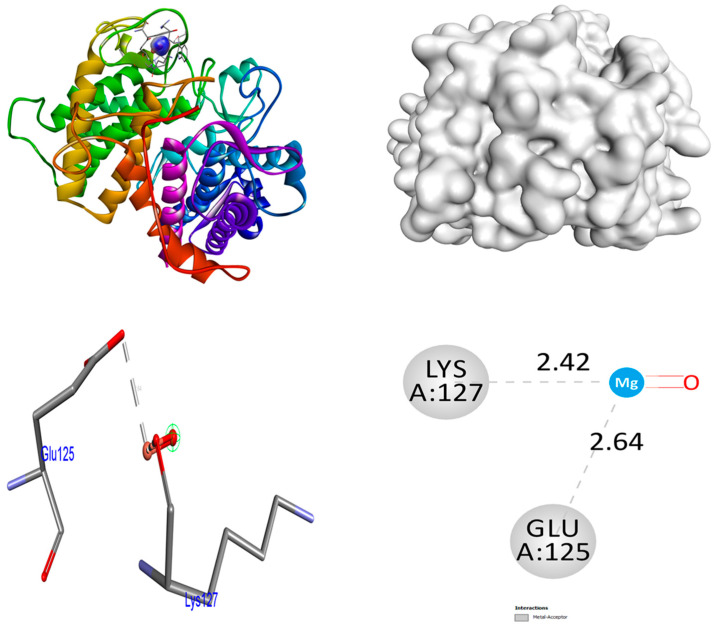
Interactions of MgO NPs inside the binding cavity of 2F6D.

**Figure 7:**
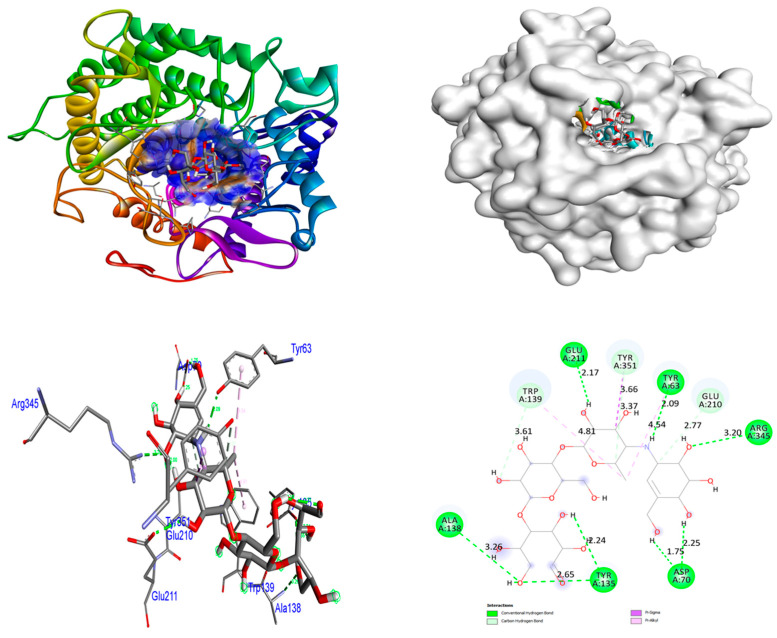
Interactions of control acarbose inside the binding cavity of 2F6D.

In order to obtain an additional inclusive grasp of the possible mechanisms of biological activities, docking investigations were undertaken. This study employed the crystalline arrangement of glucoamylase with acarbose (PDB ID: 2F6D) for the α-glucosidase inhibitory action. The analysis of the docking interactions between the ZrO_2_ and MgO NPs and the control acarbose and protein 2F6D was conducted using the Autodock Vina tool. The binding affinities of ZrO_2_ NPs are higher (−8.5 kcal/mol) than control acarbose (−6.6 kcal/mol) in the 2F6D protein. At the receptor 2F6D, the ZrO_2_ NPs do not form any hydrogen bonds. The amino acids Trp67 and Asp70 facilitated metal–acceptor interactions. The binding affinities of MgO NPs are higher (−9.2 kcal/mol) than control acarbose (−6.6 kcal/mol) in the 2F6D protein. At the receptor 2F6D, the MgO NPs do not form any hydrogen bonds. The amino acids Glu125 and Lys127 facilitated metal–acceptor interactions. At the 2F6D receptor, the control acarbose establishes eight hydrogen bond connections. The amino acid residues Tyr63 (2.09 Å), Asp70 (2.25 and 1.75 Å), Tyr135 (2.65 and 2.24 Å), Ala138 (3.26 Å), Glu211 (2.17 Å), and Arg345 (3.20 Å) were involved in hydrogen bonding associations. Distinct hydrophobic interactions were observed among the amino acid residues Trp139, Glu210, and Tyr351. The hydrophobic and hydrogen bonding contacts between amino acid residues in the 2F6D protein and ZrO_2_ and MgO NPs, and control acarbose are depicted in Figure 5, Figure 6 and Figure 7. The results were summarized in Table 5.

#### 4.1.2. α-Amylase Inhibition

**Figure 8:**
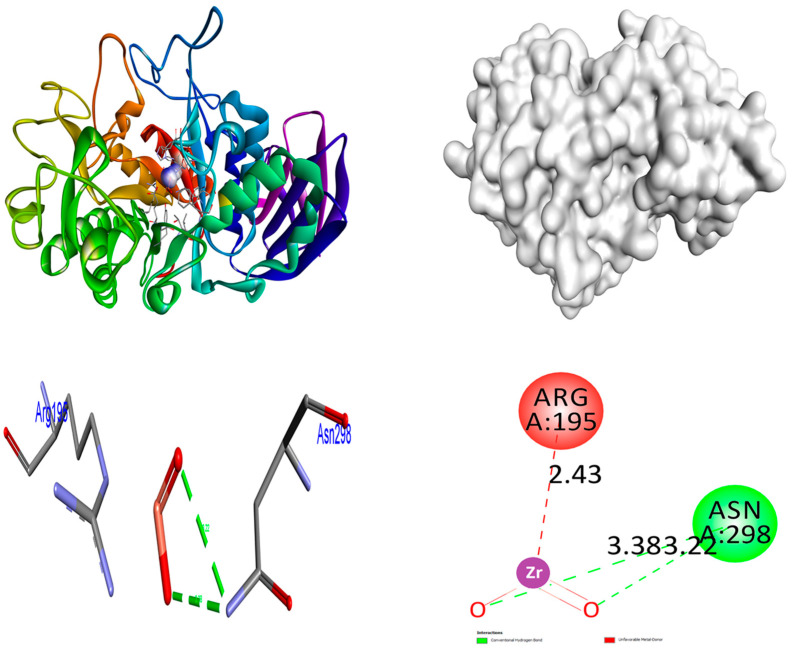
Interactions of ZrO_2_ NPs inside the binding pocket of 2QV4.

**Figure 9:**
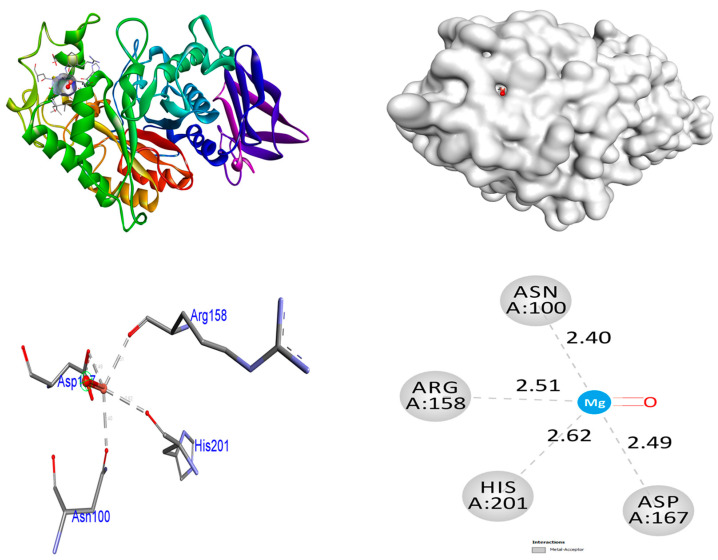
Interactions of MgO NPs inside the binding pocket of 2QV4.

**Figure 10:**
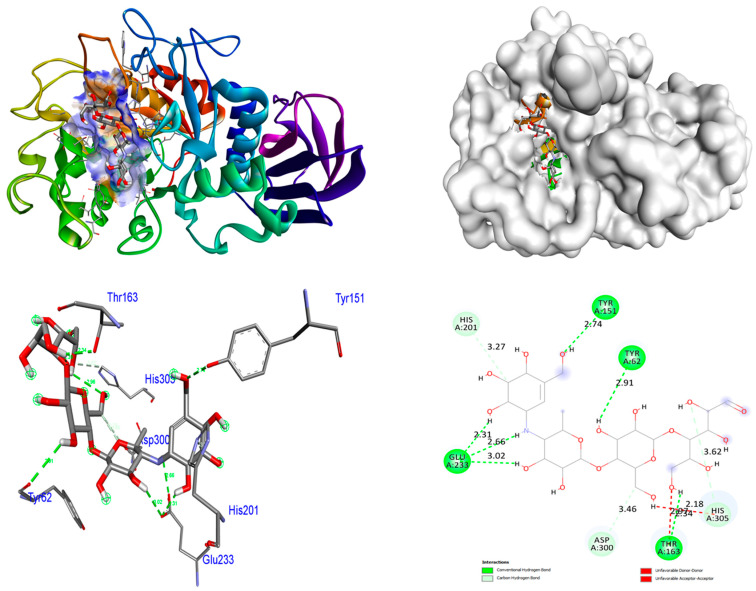
Interactions of control acarbose inside the binding pocket of 2QV4.

The present work utilized the crystalline arrangement of human pancreatic α-amylase, which was complexed with acarbose and nitrile (PDB ID: 2QV4), in order to conduct an α-amylase inhibition experiment on antidiabetic enzymes. The MgO NPs demonstrate superior binding affinities (−9.5 kcal/mol) in comparison to the control acarbose (binding affinity: −7.3 kcal/mol). MgO NPs do not form any hydrogen bonds with protein 2QV4. The amino acid residues Asn100, Arg158, Asp167, and His201 promoted metal–acceptor interactions. Among the 2QV4 proteins, ZrO_2_ NPs demonstrate superior binding affinities (−8.9 kcal/mol) in comparison to the control acarbose (binding affinity: −7.3 kcal/mol. ZrO_2_ NPs form two hydrogen bonds with protein 2QV4. The hydrogen bonding contact involving the amino acid residue Asn298 was found to have a bond length of 3.38 and 3.22 Å, respectively. The amino acid residue Arg195 promoted hydrophobic interactions. Six hydrogen bonds are fashioned amongst the control acarbose molecule and the target 2QV4. A hydrogen bonding interaction was involved among the amino acid residues Tyr62 (bond length: 2.91 Å), Tyr151 (bond length: 2.74 Å), Thr162 (bond length: 2.34 Å), and Glu233 (bond lengths: 3.02, 2.66, and 2.31 Å). The His201, Asp300, and His305 amino acid residues promoted hydrophobic relations. Hydrophobic and hydrogen bonding connections amongst amino acid residues in the 2QV4 protein and ZrO_2_ and MgO NPs and control acarbose are illustrated in Figure 8, Figure 9 and Figure 10. The findings are represented in Table 6.

### 4.2. Antioxidant

**Figure 11:**
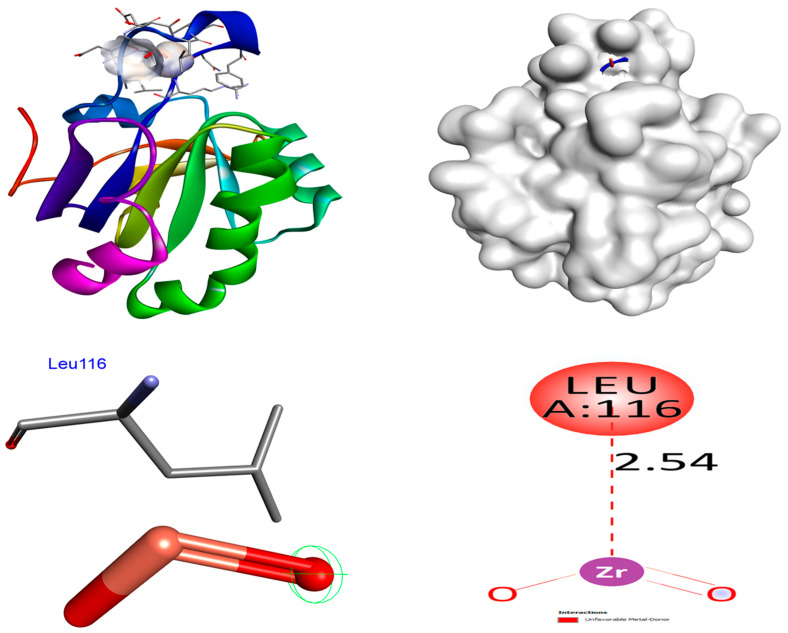
Interactions of ZrO_2_ NPs inside the binding pocket of 3MNG.

**Figure 12:**
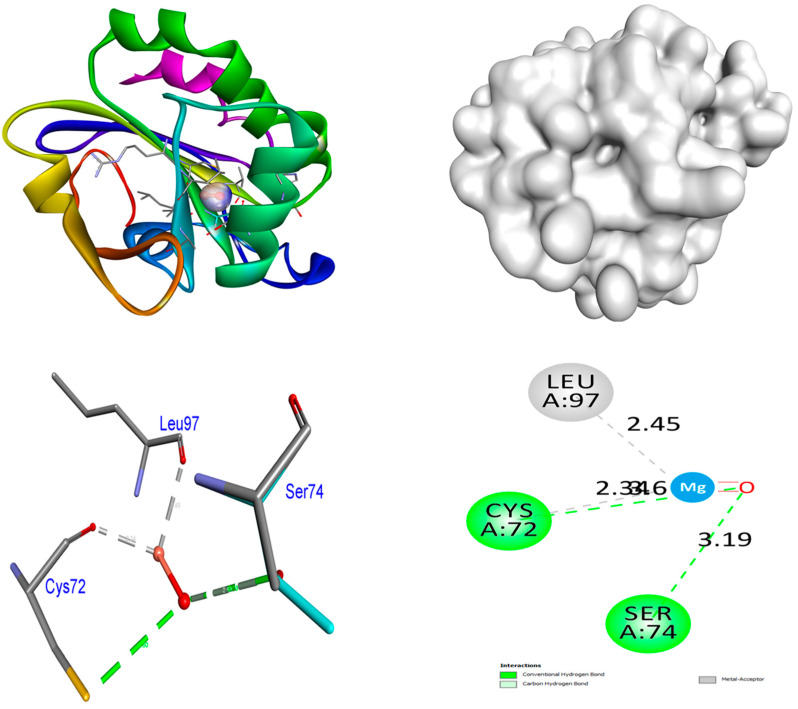
Interactions of MgO NPs inside the binding pocket of 3MNG.

**Figure 13:**
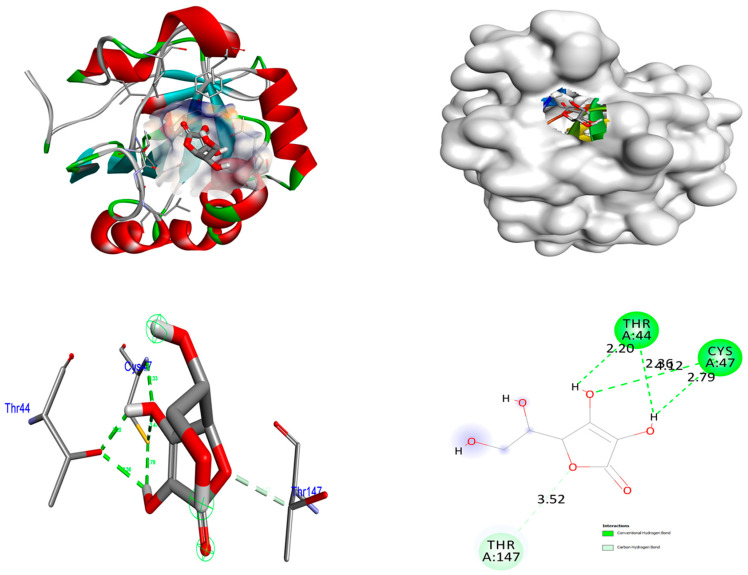
Interactions of control Ascorbic acid inside the binding pocket of 3MNG protein.

The optimal target for antioxidant studies is the human antioxidant enzyme’s crystal structure in association with the modest inhibitor DTT (PDB: 3MNG). The binding affinities of ZrO_2_ NPs are higher (−7.4 kcal/mol) than control ascorbic acid (−4.3 kcal/mol) in the 3MNG protein. No hydrogen bonds are formed between ZrO_2_ NPs and the protein 3MNG. The Leu116 amino acid residue promoted hydrophobic interactions. The binding affinities of MgO NPs are higher (−8.3 kcal/mol) than control ascorbic acid (−4.3 kcal/mol) in the 3MNG protein. Two hydrogen bonds are formed between MgO NPs and the protein 3MNG. The residues of amino acids Cys72 and Ser74 were involved in hydrogen bonding. The Leu97 amino acid residue promoted hydrophobic interactions. The control compound, ascorbic acid, establishes four hydrogen bond contacts with the protein 3MNG. In hydrogen bonding connections, the amino acid residues Thr44 (bond lengths: 2.36 and 2.20 Å) and Cys47 (bond lengths: 4.12 and 2.79 Å) were involved. Distinct hydrophobic connections were observed in the amino acid residue Thr147. The hydrophobic and hydrogen bonding relations between amino acid residues in the 3MNG protein and ZrO_2_ and MgO NPs and control ascorbic acid are depicted in Figure 10, Figure 11, Figure 12 and Figure 13. The findings suggest that MgO NPs demonstrate a significantly greater ability to suppress antidiabetic and antioxidant characteristics compared to the control compounds acarbose and ascorbic acid. Our in silico analysis indicates that both MgO and ZrO_2_ nanoparticle models can occupy catalytically relevant pockets of antioxidant and antidiabetic proteins with energetically favorable poses. These findings are consistent with prior MgO docking reports that linked predicted binding to observed α-glucosidase/α-amylase inhibitory activity, such as Mazhar et al. [42], who reported −7.8 to −8.4 kcal/mol binding energies correlating with in vitro IC_50_ values, and Negi et al. [43], who found −8.5 kcal/mol for Ag/MgO nanocomposites against α-amylase. Similarly, Al-Harbi et al. [44] and Shahid et al. [45] documented MgO docking to catalase, peroxidase, and superoxide dismutase with strong binding scores that paralleled high antioxidant activity. Our work also expands the limited set of zirconia docking studies—most of which have targeted microbial or non-enzymatic proteins [46,47,48]—by demonstrating potential interactions with mammalian antioxidant/antidiabetic targets. Given the diversity of modelling choices in the literature (NP representation, docking engine, solvation, and receptor flexibility), our comparative docking results should be interpreted alongside experimental enzyme inhibition (see Table 7).

## 5. Conclusions

The present research deals with the biogenesis of MgO and ZrO_2_ NPs, utilizing *Solanum trilobatum* as a template. The formation of nanostructures was confirmed using various spectroscopic and microscopic analyses. The synthesized nanomedicines were further assessed for oxidative stress and hypoglycemia. Analytical studies indicate that MgO and ZrO_2_ NPs measure 60–80 and 15–30 nm in diameter and possess a circular morphology, ensuring the MgO and ZrO_2_ NPs are more appropriate for biotic usage. This study encompasses the domains of nanotechnology and phytochemistry. The uses of *Solanum trilobatum*-derived green-generated MgO and ZrO_2_ NPs demonstrated optimal antioxidant and hypoglycemic efficacy. The reaction of MgO and ZrO_2_ NPs is contingent upon dosage, with 500 μg/mL and 1000 μg/mL established as effective concentrations. The results of the DPPH scavenging test and the FRAP assay exceeded those of ascorbic acid. The inhibitory efficacy of α-amylase and α-glucosidase was comparable to that of acarbose. MgO NPs demonstrated excellent antidiabetic potential through the inhibition of α-amylase and α-glucosidase enzymes compared to the standard acarbose. The phyto-constituents of *Solanum trilobatum* may synergistically enhance the antioxidant and hypoglycemic potential of MgO and ZrO_2_ NPs. Further, in silico molecular docking studies were performed to drive the inhibiting mechanism of MgO and ZrO_2_ NPs towards antioxidant and antidiabetic enzymes. In silico molecular docking studies support the in vitro study, and both the nanomedicines bind to the target proteins through various hydrophobic interactions. The in vitro test findings elucidate the anti-diabetic and antioxidant properties of green-produced MgO and ZrO_2_ NPs for prospective in vivo and clinical studies, in addition to pharmaceutical development.

## Figures and Tables

**Table 1:** DPPH radical scavenging action.

Compound	Scavenging Activity (%)				IC_50_ (μM)
	1000 (μM)	500 (μM)	250 (μM)	125 (μM)	
**Plant extract**	161.7 ± 2.8	66.2 ± 1.4	42.4 ± 0.9	21.8 ± 1.3	323.49
**ZrO_2_ NPs**	325.0 ± 1.6	121.7 ± 2.3	83.4 ± 1.4	32.4 ± 2.9	191.96
**MgO NPs**	327.0 ± 1.7	123.8 ± 2.3	85.6 ± 1.6	34.6 ± 2.4	185.23
**Ascorbic acid**	312.1 ± 0.5	118.4 ± 0.2	63.4 ± 0.9	42.7 ± 1.4	201.75

**Table 2:** FRAP antioxidant assay.

Compound	Scavenging Activity (%)				IC_50_ (μM)
	1000 (μM)	500 (μM)	250 (μM)	125 (μM)	
**Plant extract**	85.3 ± 1.5	47.4 ± 2.6	25.7 ± 1.3	11.3 ± 2.4	560.08
**ZrO_2_ NPs**	191.5 ± 1.7	121.6 ± 2.3	71.7 ± 1.8	31.3 ± 1.6	161.86
**MgO NPs**	194.8 ± 2.4	124.9 ± 1.3	75.0 ± 2.7	34.6 ± 1.2	143.12
**Ascorbic acid**	211.7 ± 1.3	111.3 ± 2.5	55.4 ± 1.2	27.6 ± 2.5	223.51

**Table 3:** α-Glucosidase inhibition assay.

Compound	Inhibition Rate (%)				IC_50_ (μM)
	1000 (μM)	500 (μM)	250 (μM)	125 (μM)	
**Plant extract**	83.6 ± 1.6	53.7 ± 2.4	39.3 ± 1.2	21.5 ± 2.5	475.92
**ZrO_2_ NPs**	207.5 ± 2.3	133.4 ± 1.7	69.3 ± 0.8	33.9 ± 1.6	155.76
**MgO NPs**	210.8 ± 2.8	136.7 ± 1.4	72.6 ± 2.3	37.2 ± 1.2	138.84
**Acarbose**	185.6 ± 0.4	87.3 ± 0.5	49.4 ± 2.3	29.6 ± 0.2	256.37

**Table 4:** α-amylase inhibition action.

Compound	Inhibition Rate (%)				IC_50_ (μM)
	1000 (μM)	500 (μM)	250 (μM)	125 (μM)	
**Plant extract**	121.5 ± 1.3	52.9 ± 2.5	43.7 ± 1.6	21.4 ± 0.5	378.78
**ZrO_2_ NPs**	211.5 ± 2.6	141.7 ± 1.6	73.6 ± 0.5	31.5 ± 1.7	145.95
**MgO NPs**	214.8 ± 1.2	145.0 ± 2.1	76.9 ± 1.6	34.8 ± 2.3	129.45
**Acarbose**	211.3 ± 0.6	121.7 ± 0.3	47.8 ± 1.5	27.4 ± 2.6	225.65

**Table 5:** Molecular docking interaction of ZrO_2_ and MgO NPs against protein 2F6D.

Compounds	Glucoamylase (PDB ID: 2F6D)		
	Binding Affinity (kcal/mol)	No. of H-Bonds	H-Bonding Residues
**ZrO_2_ NPs**	−8.5	-	-
**MgO NPs**	−9.2	-	-
**Acarbose**	−6.6	8	Tyr63, Asp70, Tyr135, Ala138, Glu211, Arg345

**Table 6:** Molecular docking interaction of ZrO_2_ and MgO NPs against protein 2QV4.

Compounds	Human Pancreatic α-Amylase (PDB ID: 2QV4)		
	Binding Affinity (kcal/mol)	No. of H-Bonds	H-Bonding Residues
**ZrO_2_ NPs**	−8.9	2	Asn298
MgO	−9.5	-	-
**Acarbose**	−7.3	6	Tyr62, Tyr151, Thr163, Glu233

**Table 7:** Molecular docking interaction of ZrO_2_ and MgO NPs against protein 3MNG.

Compounds	Human Antioxidant Enzyme (PDB: 3MNG)		
	Binding Affinity (kcal/mol)	No. of H-Bonds	H-Bonding Residues
**ZrO_2_ NPs**	−7.4	-	-
MgO NPs	−8.3	2	Cys72, Ser74
**Ascorbic acid**	−4.3	4	Thr44, Cys47

## Data Availability

The original contributions presented in this study are included in the article. Further inquiries can be directed to the corresponding authors.
